# Acute Cognitive Effects of Brief Physical Activity Breaks After Lecture-Based Academic Activity in Undergraduate University Students: A Randomized Crossover Study

**DOI:** 10.3390/healthcare14132010

**Published:** 2026-07-06

**Authors:** Ilaria Pepe, Alessandro Petrelli, Luca Poli, Francesco Fischetti, Stefania Cataldi, Gianpiero Greco

**Affiliations:** 1 Department of Translational Biomedicine and Neuroscience (DiBraiN), University of Bari “Aldo Moro”, 70124 Bari, Italy; ilaria.pepe@uniba.it (I.P.); alessandro.petrelli@uniba.it (A.P.); gianpiero.greco@uniba.it (G.G.); 2 Department of Neurosciences, Biomedicine and Movement Sciences, University of Verona, 37129 Verona, Italy; 3 Department of Education and Sport Sciences, Pegaso Telematic University, 80143 Naples, Italy; stefania.cataldi@unipegaso.it

**Keywords:** active breaks, outdoor physical activity, exergaming, attentional processes, executive functioning, higher education, cognitive fatigue, health promotion

## Abstract

**Background**: Prolonged sitting during lecture-based academic activities may be accompanied by sustained attentional engagement and cognitive fatigue, with potential consequences for cognitive efficiency and broader psychological functioning. Physical activity breaks (PABs) represent a feasible strategy to interrupt sitting time within university timetables, yet evidence in higher-education settings remains limited, particularly regarding modality-specific effects. This randomized crossover study examined the acute effects of a 10 min PAB on attentional and executive functioning in undergraduate students and compared an outdoor physical activity break (OPAB) with an exergame-based PAB (PABEx) versus a no-break control (NPAB). **Methods**: Forty-two undergraduate students (26 males, 16 females; mean age = 22.78 ± 5.84 years) completed three weekly conditions in randomized order following two consecutive hours of seated lectures: NPAB (seated rest), OPAB (2 min warm-up, 6 min light-to-moderate walking, 2 min cool-down), and PABEx (2 min warm-up, 6 min Fruit Ninja Kinect, 2 min cool-down). Cognitive performance was assessed immediately after each condition using the Trail Making Test A-B (TMT A-B) and the Stroop Color-Word Test (SCWT). **Results**: Significant condition effects were found for TMT-A (χ^2^ = 53.976, *p* < 0.001), TMT-B (χ^2^ = 44.635, *p* < 0.001), TMT B-A (χ^2^ = 10.841, *p* = 0.004), SCWT interference time (χ^2^ = 44.714, *p* < 0.001), and SCWT interference errors (χ^2^ = 23.211, *p* < 0.001). Post-hoc tests showed that both OPAB and PABEx were associated with better performance on TMT-A, TMT-B, and SCWT interference time versus NPAB (all Benjamini–Hochberg-adjusted *p* < 0.001); PABEx was associated with better TMT-A performance than OPAB (Benjamini–Hochberg-adjusted *p* = 0.047). TMT B-A decreased only for OPAB versus NPAB (Benjamini–Hochberg-adjusted *p* = 0.009). SCWT interference errors were lower for OPAB versus NPAB (Benjamini–Hochberg-adjusted *p* < 0.001) and for PABEx versus NPAB (Benjamini–Hochberg-adjusted *p* = 0.012). **Conclusions**: A 10 min PAB implemented immediately after a lecture-based academic activity was associated with more favorable post-condition attentional and executive performance in undergraduate students compared with a passive no-break condition. OPAB and PABEx yielded broadly comparable benefits across executive outcomes, whereas PABEx showed an additional advantage for TMT-A, suggesting a possible modality-specific effect on processing speed and visuoperceptual tracking. These findings support the integration of brief active breaks into university schedules as a pragmatic strategy to promote post-lecture cognitive efficiency during academically demanding periods. Trial registration: ClinicalTrials.gov, NCT07624084; retrospectively registered on 28 May 2026.

## 1. Introduction

Concurrent physiological, psychological, and social changes throughout early adulthood may impact health-related behaviors, such as physical activity [1]. Consistent evidence suggests that sedentary behavior is common in undergraduate populations and typically increases across the transition from late adolescence to early adulthood [2,3]. Sedentary behavior is defined as any waking behavior performed while sitting, reclining, or lying that entails an energy expenditure of ≤1.5 METs [4].

Alongside these high sedentary levels, adherence to physical activity recommendations is low; only a limited proportion of undergraduate students achieve the recommended amounts of moderate-to-vigorous physical activity (MVPA), and most report no regular engagement in structured or leisure-time physical activity [5]. Evidence suggests that prolonged sedentary behavior is associated with adverse health outcomes [3,6,7], and this relationship persists even when recommended physical activity levels are met. Accordingly, sedentary behavior and insufficient physical activity are increasingly regarded as separate, independent risk factors for poor health, including cognitive health [8,9], with plausible downstream consequences for students’ learning processes and, ultimately, their academic performance [10].

To reduce sedentary behavior and its related health risks, physical activity guidelines across many countries recommend minimizing prolonged sitting and regularly breaking up extended periods of sedentary time whenever feasible [11]. Incorporating active breaks, brief, structured bouts of physical activity implemented during or between classes, within the academic schedule, is a feasible strategy and has been associated with improvements in students’ attention, learning, and cognitive performance [12,13]. Cognitive performance refers to cognitive/executive functioning, defined as a set of higher-order control processes that govern thought and behavior [14], which encompasses multiple mental abilities, including reasoning, memory, problem solving, decision making, attentional control, and learning [15].

Recent studies indicate that embedding physical activity within instructional time through physical activity breaks (PABs) may yield benefits for selective attention, academic achievement, mental well-being, and enjoyment [16,17,18]. Converging evidence further suggests that breaking up prolonged sitting with brief bouts of physical activity can attenuate the potential detrimental effects of sedentary behavior on cognition [19], both acutely (i.e., following a single break) and when implemented repeatedly over time (i.e., across multiple sessions) [19,20].

In response, several authors have sought to delineate the “optimal” characteristics of PABs, namely duration (minutes), modality, and intensity (light, moderate, vigorous), as these parameters may moderate cognitive responses. However, the literature remains heterogeneous and does not yet converge on an “optimal” prescription. With respect to duration, available evidence has examined a relatively broad range of break durations, generally spanning from approximately 5 to 20 min, with partly inconsistent findings across studies. Shorter bouts have been associated with favorable cognitive responses in some investigations [21], whereas other studies suggest that breaks of approximately 10 min may be sufficient to elicit measurable changes in alertness, at least in younger samples [22,23]. Thus, rather than identifying a single optimal duration, current evidence supports the relevance of brief active breaks within this time range, while leaving the precise dose-response relationship unresolved. Intensity may also influence the cognitive responses elicited by PABs. Previous studies suggest that light-to-moderate activity may be sufficient to support short-term attentional and executive performance, whereas very low or excessively demanding activity may produce smaller or less consistent cognitive effects [24,25,26]. However, the optimal intensity of brief active breaks remains difficult to define, as cognitive responses may vary according to population characteristics, task demands, exercise modality, and contextual factors.

Evidence on the role of modality is comparatively limited. Outdoor and classroom-based active breaks have been associated with improvements in well-being, sustained attention, task behavior, and academic-related outcomes in school and workplace contexts [22,27,28]. Evidence from higher-education settings is also emerging, with studies suggesting that classroom movement breaks and physically active learning may reduce sedentary behavior and fatigue, while supporting focus, attention, cognitive functioning, mental well-being, and academic-related outcomes in university students [12,28]. However, fewer studies have examined whether different active-break modalities elicit distinct acute cognitive responses after lecture-based academic activity.

Exergaming, a portmanteau of “exercise” and “gaming” [29], is defined as physically active video gaming that is both physically demanding and motivational [30]. It provides a structured and replicable task format, while still allowing individual variability in movement execution, response strategies, and familiarity with game-based tasks [29].

Studies indicate that exergame-based bouts are feasible and may acutely enhance executive functioning and broader cognitive performance [31,32,33], with related work suggesting potential benefits of virtual-reality-augmented aerobic exercise for cognitive outcomes [34].

The purpose of this study was to examine whether PABs are associated with more favorable post-condition cognitive functioning in university students, an area that remains understudied compared to primary school populations. Cognitive functioning was considered the overall outcome domain of the study and was assessed through complementary indices of attentional and executive performance. The study was designed as a randomized crossover exploratory trial aimed at comparing acute post-condition cognitive performance after three different break modalities implemented following a lecture-based academic activity. Specifically, the study examined whether, within the same participants, post-condition attentional and executive performance differed after an outdoor PAB and an exergaming-based PAB compared with a passive no-break control condition. We hypothesized that both PAB conditions would be associated with better attentional and executive performance than the control condition, while possible differences between the outdoor and exergaming modalities were examined in an exploratory manner.

## 2. Materials and Methods

### 2.1. Participants and Study Design

Between April and May 2025 (second semester of the academic year), an exploratory randomized controlled crossover study was conducted to examine the effects of physical activity breaks (PABs) on attentional and executive functions in university students.

A total of 42 undergraduate university students, 26 males and 16 females (Mage = 22.78, SD = 5.84), were recruited at the University of Bari Aldo Moro (Bari, Italy) during the teaching period and completed the study procedures. Prior to participation, students received a standardized explanation of the study aims and procedures and provided written informed consent.

Students were eligible if they were (i) enrolled full-time, (ii) 18–35 years old at the time of data collection, and (iii) physically able to stand and perform brief bouts of physical activity safely. Exclusion criteria comprised musculoskeletal disorders, current or recent lower-limb injuries, and acute or chronic medical conditions that could limit safe participation in light-to-moderate physical activity. Students were also excluded if they were unable to comply with the study protocol (e.g., attendance at scheduled sessions and completion of the cognitive assessments).

An a priori power analysis was performed using G*Power version 3.1 [35] to estimate the minimum sample size required to detect within-subject effects in the planned repeated-measures design. The analysis was specified as F tests (ANOVA: repeated measures, within factors) with an assumed medium effect size (f = 0.25), α = 0.05, and power (1-β) = 0.90. Model parameters were set to three repeated measurements, an expected correlation among repeated measures of 0.50, and a nonsphericity correction of ε = 1. This yielded a required total sample size of n= 36 (actual power = 0.906). The final sample (n = 42) exceeded this threshold. Although the final inferential analyses were conducted using non-parametric tests due to assumption violations, the a priori power analysis was retained as a planning estimate for the repeated-measures study design.

All data were managed in accordance with the American Psychological Association guidelines to ensure anonymity and confidentiality. The study was conducted in accordance with the ethical principles of the Declaration of Helsinki for research involving human participants, approved by the Ethics Committee for Research of the University of Bari Aldo Moro (approval reference: 116866; 18 April 2025), and registered at ClinicalTrials.gov (ID: NCT07624084).

### 2.2. Setting and Procedure

All study procedures were embedded within routine university teaching weeks and scheduled in the morning (09:00–12:00). Each participant completed three experimental sessions, one per week, over three consecutive weeks; sessions were separated by a 7-day interval that served as the washout period. On each testing day, participants first attended two consecutive hours of scheduled university lectures (i.e., standard didactic instruction delivered in a lecture-based format), during which they remained seated and engaged in typical academic activities such as listening, note-taking, and following course-related content, with no planned movement opportunities. Immediately after the lecture block, participants completed the condition assigned for that week: (i) no physical activity break (NPAB), (ii) outdoor physical activity break (OPAB), or (iii) Exergame-based physical activity break (PABEx). The order of the three conditions was randomized and counterbalanced across the three consecutive weeks to reduce order effects, carry-over influences, and repeated-testing bias. Participants were assigned to one of the six possible condition sequences using a computer-generated randomization list prepared before data collection. Sequence allocation was concealed from the researcher administering the cognitive assessments, who remained blinded to the condition completed by each participant during that week. Sessions were separated by a 7-day washout interval, which was considered adequate given the acute nature of the intervention and the short duration of each condition.

**Figure 1:**
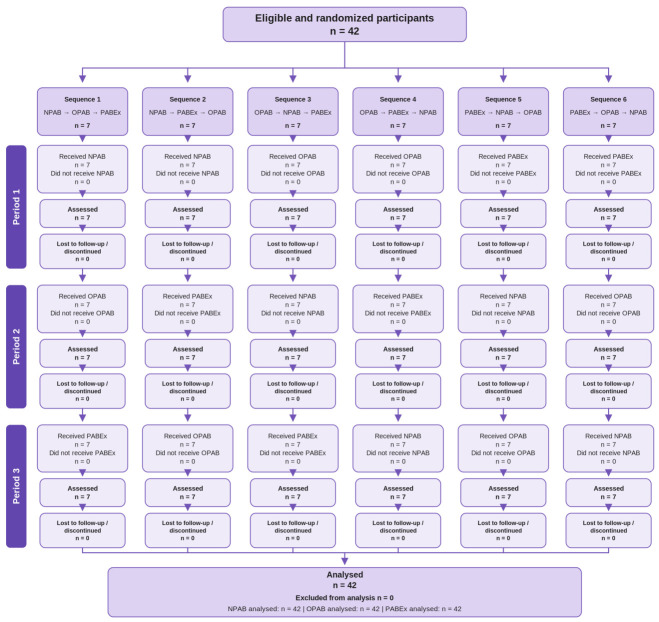
CONSORT flow diagram for the randomized crossover trial. Forty-two undergraduate students were randomized to one of the six possible sequences of the three experimental conditions and completed all three experimental periods. NPAB = No Physical Activity Break; OPAB = Outdoor Physical Activity Break; PABEx = Physical Activity Break with Exergame. No participants were lost to follow-up, discontinued the intervention, or were excluded from the final analysis.

The participant flow through enrollment, randomization, crossover periods, follow-up, and analysis is shown in Figure 1. The completed CONSORT checklist for randomized crossover trials is available as Supplementary Material (Table S1).

To ensure procedural standardization, all experimental activities were conducted within the same facility and followed a standardized timeline. To reduce contextual variability, the three conditions were carried out in predetermined areas. OPAB was conducted in a specific outdoor setting, PABEx was performed in a dedicated indoor room equipped for the exergame protocol, and NPAB involved supervised quiet seated rest. Cognitive testing was administered immediately after completion of each condition in a quiet, dedicated testing room, under controlled environmental conditions (minimal noise, limited foot traffic, and reduced visual distractions), using the same testing equipment and a standardized instruction script at every session. All assessments were administered by a trained psychology researcher who remained blinded to the condition completed by each participant during that week. The morning test window was selected to limit diurnal variability in attentional and executive outcomes and to avoid peak outdoor temperatures during OPAB sessions, which were carried out under mild spring environmental conditions, with ambient temperature and relative humidity averaging approximately 18 °C and 70%, respectively.

To further minimize the influence of extraneous factors on cognitive outcomes, participants were instructed to maintain their usual sleep routine, refrain from vigorous physical activity during the 24 h preceding each testing session, and avoid caffeine intake during the same period. They were also asked to arrive having eaten their usual breakfast and to avoid introducing atypical dietary or stimulant habits on testing days.

### 2.3. Physical Activity Break Interventions

Participants were instructed to refrain from vigorous physical activity during the 24 h preceding each experimental session, as described above. After completing two consecutive hours of scheduled university lectures, participants engaged in the condition assigned for that week. All conditions lasted 10 min and were administered immediately after the lecture period to ensure consistency across sessions.

#### 2.3.1. No Physical Activity Break (NPAB)

The NPAB condition served as the control condition. Participants paused their academic activities for 10 min while remaining seated at their workstations, representing a traditional passive rest break. No structured movement or physical activity was prescribed, and participants were instructed to rest quietly.

#### 2.3.2. Outdoor Physical Activity Break (OPAB)

The OPAB condition consisted of a standardized 10 min outdoor protocol structured into three sequential phases: (i) Warm-up (2 min). Participants performed low-intensity dynamic movements designed to gradually increase physiological arousal and prepare major joints for locomotor activity (e.g., joint mobilization of the ankles, knees, hips, and shoulders, combined with light marching in place). (ii) Walking phase (6 min). Participants completed a walk along a predefined outdoor route at an intended pace of approximately 4.5 km·h^−1^, selected to provide a light-to-moderate aerobic stimulus and to ensure procedural reproducibility across participants and sessions. However, exercise intensity was not objectively monitored through heart rate or rating of perceived exertion. (iii) Cool-down (2 min). The session concluded with relaxation and gentle stretching exercises aimed at progressively reducing activation and facilitating recovery, primarily targeting lower-limb muscle groups and breathing regulation.

#### 2.3.3. Physical Activity Break with Exergame (PABEx)

The PABEx condition was designed to provide an active break through a non-immersive exergaming modality, while maintaining the same overall duration and temporal structure as OPAB. The 10 min session was organized as follows: (i) Warm-up (2 min). Participants completed the same dynamic warm-up used in the OPAB condition to standardize pre-activity, physiological activation. (ii) Exergame phase (6 min). The exergame used in the study was Fruit Ninja Kinect, a non-immersive active video game. Participants were instructed to stand facing a television screen at approximately 1.5–2.0 m and to use the Kinect motion-sensing controller to interact with the game. Fruit Ninja requires players to perform repeated, rapid arm and hand movements to wield virtual swords and slice fruit that appears on the screen. Gameplay involves continuous upper-limb movement, visuomotor coordination, rapid stimulus detection, and response execution, thereby combining physical engagement with sustained attentional and executive demands. (iii) Cool-down (2 min). The session concluded with the same relaxation and gentle stretching exercises used in OPAB to standardize post-activity recovery across active conditions. Although the duration, game, setting, and instructions were standardized, the physiological intensity of the exergame condition was not objectively monitored and may have varied according to individual movement amplitude, response frequency, and familiarity with exergaming.

Across all conditions, session duration, sequencing, supervision, and the timing of subsequent cognitive testing were kept constant to isolate the effects of break modality (passive rest, outdoor walking, or exergame-based activity) on attentional and executive functioning.

### 2.4. Cognitive Assessment

#### 2.4.1. Trail Making Test A and B (TMT-A and TMT-B)

The Trail Making Test (TMT) [36] is a widely used neuropsychological measure of attention and executive functioning, providing indices of visual search and scanning, information processing speed, and cognitive flexibility [37]. In TMT-A, participants are required to draw a continuous line connecting 25 numbered circles in ascending order as quickly and accurately as possible. In TMT-B, the task is more demanding because participants must connect circles by alternating between numbers and letters in sequence (e.g., 1-A-2–B-3-C), thereby placing greater demands on set-shifting and mental flexibility. For both parts, performance is quantified as completion time, with shorter times indicating better performance.

#### 2.4.2. The Stroop Color and Word Test (SCWT)

The Stroop Color and Word Test (SCWT) was used to assess interference control and response inhibition, core components of executive functioning [38]. The SCWT comprises three trials. In the first (Word) trial, participants read aloud lists of color names (e.g., red, green, blue) as quickly as possible. In the second (Color) trial, they named the ink color of non-linguistic stimuli (e.g., colored dots) presented in the same set of colors. In the third (Color-Word) trial, color words are printed in incongruent ink colors (e.g., the word RED printed in blue ink), and participants must name the ink color while suppressing the prepotent tendency to read the word. This incongruent condition provides the primary index of susceptibility to interference (the Stroop effect), reflecting the efficiency of inhibitory control. For the present study, interference sensitivity was quantified using both time-based and accuracy-based indices, derived respectively from the time required to complete the trials (interference/time) and the number of errors committed (interference/errors).

### 2.5. Statistical Analysis

All statistical analyses were performed using IBM SPSS Statistics version 26.0 (IBM Corp., Armonk, NY, USA). Prior to inferential analyses, raw scores obtained from all neuropsychological measures were adjusted for age and educational level according to the normative correction procedures provided for each test. Only these age- and education-adjusted scores were used in subsequent analyses.

Preliminary assumption checks were conducted to evaluate the suitability of parametric repeated-measures analyses. Normality of score distributions for each condition was assessed using the Shapiro-Wilk test, complemented by visual inspection of histograms and Q-Q plots. In addition, the assumption of sphericity required for repeated-measures ANOVA was evaluated using Mauchly’s test. These preliminary analyses indicated that parametric assumptions were not consistently satisfied across outcome measures. Differences across the three within-subject conditions (NPAB, OPAB, PABEx) were therefore tested using the Friedman test, the non-parametric alternative to a one-way repeated-measures ANOVA. Accordingly, five omnibus Friedman tests were conducted, one for each cognitive index: TMT-A, TMT-B, TMT B-A, Stroop interference time, and Stroop interference errors. When the omnibus test was significant, three pairwise post-hoc comparisons were performed for each outcome using Conover tests: NPAB versus OPAB, NPAB versus PABEx, and OPAB versus PABEx. Because all five omnibus tests reached statistical significance, a total of fifteen post-hoc pairwise comparisons were conducted. Kendall’s W was used as the effect size for Friedman tests and interpreted using conventional thresholds: small ≈ 0.10, moderate ≈ 0.30, and large ≈ 0.50.

Multiplicity was addressed at two levels. First, the Benjamini–Hochberg false discovery rate procedure was applied across the five omnibus Friedman tests. Second, the fifteen Conover post-hoc pairwise comparisons were interpreted after Benjamini–Hochberg correction across the full family of pairwise contrasts.

To examine possible period effects associated with repeated cognitive testing, additional Friedman tests were conducted for each cognitive outcome by comparing scores across the three testing periods, irrespective of the experimental condition performed in each period. For each outcome, test statistics, exact *p*-values, effect sizes, and condition-specific descriptive values are reported.

## 3. Results

### 3.1. Trail Making Test

**Figure 2:**
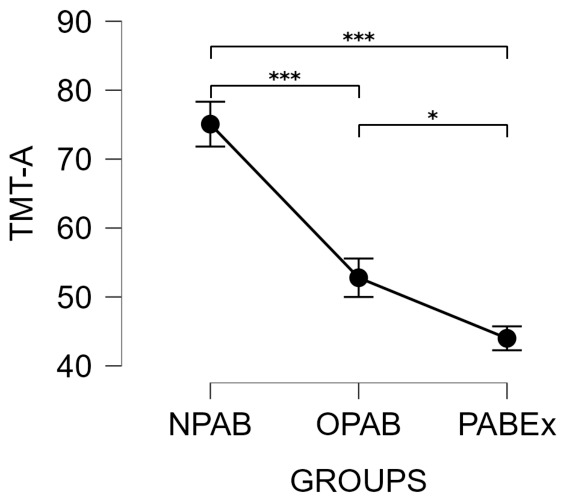
TMT-A completion time across conditions. *Notes.* Values represent means of age- and education-corrected TMT-A scores; error bars indicate ±1 SD. Lower corrected completion-time scores indicate better performance. Significance brackets and asterisks indicate Benjamini–Hochberg-adjusted Conover post-hoc comparisons (* *p* < 0.05; ** *p* < 0.01; *** *p* < 0.001). Abbreviations: TMT-A = Trail Making Test-Part A; NPAB = No Physical Activity Break; OPAB = Outdoor Physical Activity Break; PABEx = Physical Activity Break with Exergame.

Condition significantly affected TMT-A corrected scores (Friedman χ^2^(2) = 53.976, Benjamini–Hochberg-adjusted *p* < 0.001), with a large condition effect (Kendall’s W = 0.643). Mean ± SD adjusted scores were 75.07 ± 24.62 for NPAB, 52.79 ± 20.54 for OPAB, and 44.00 ± 9.13 for PABEx. Post-hoc Conover comparisons, interpreted after Benjamini–Hochberg correction across the full family of pairwise contrasts, showed significantly lower completion-time scores for OPAB compared with NPAB (Benjamini–Hochberg-adjusted *p* < 0.001) and for PABEx compared with NPAB (Benjamini–Hochberg-adjusted *p* < 0.001). PABEx also showed significantly lower completion-time scores than OPAB (Benjamini–Hochberg-adjusted *p* = 0.047) (Figure 2).

**Figure 3:**
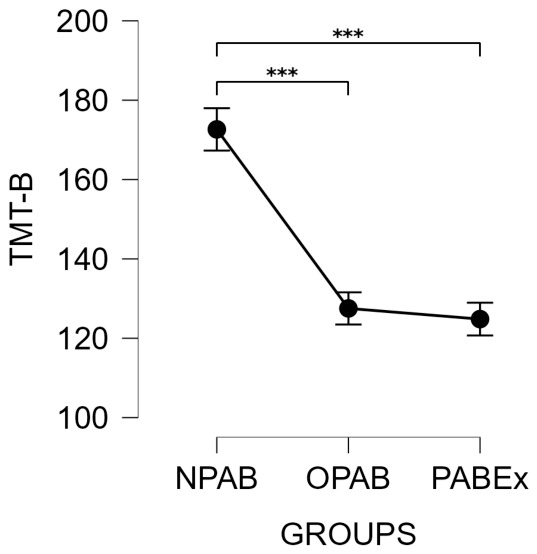
TMT-B completion time across conditions. *Notes.* Values represent means of age- and education-corrected TMT-B scores; error bars indicate ±1 SD. Lower corrected completion-time scores indicate better performance. Significance brackets and asterisks indicate Benjamini–Hochberg-adjusted Conover post-hoc comparisons (* *p* < 0.05; ** *p* < 0.01; *** *p* < 0.001). *Abbreviations*: TMT-B = Trail Making Test-Part B; NPAB = No Physical Activity Break; OPAB = Outdoor Physical Activity Break; PABEx = Physical Activity Break with Exergame.

For TMT-B, corrected scores varied significantly across conditions (Friedman χ^2^(2) = 44.635, Benjamini–Hochberg-adjusted *p* < 0.001), with a large condition effect (Kendall’s W = 0.531). Mean ± SD adjusted scores were 172.64 ± 39.88 for NPAB, 127.52 ± 31.11 for OPAB, and 124.83 ± 35.60 for PABEx. As shown in Figure 3, post-hoc Conover comparisons, interpreted after Benjamini–Hochberg correction across the full family of pairwise contrasts, showed significantly lower completion-time scores for OPAB compared with NPAB (Benjamini–Hochberg-adjusted *p* < 0.001) and for PABEx compared with NPAB (Benjamini–Hochberg-adjusted *p* < 0.001). The difference between OPAB and PABEx was not statistically significant (Benjamini–Hochberg-adjusted *p* = 0.202).

**Figure 4:**
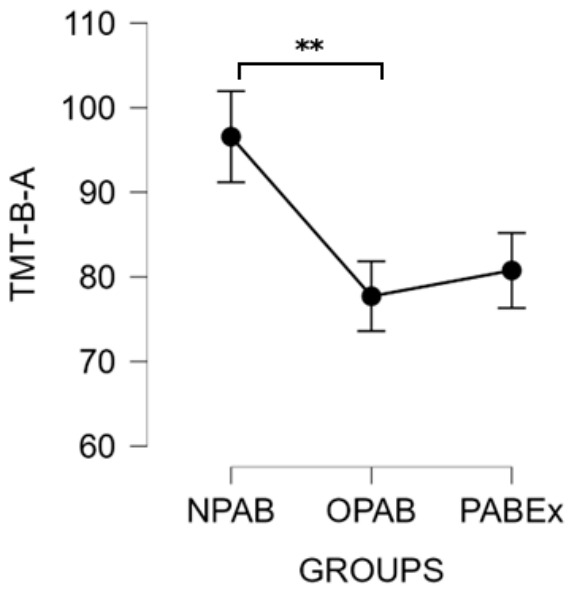
TMT B-A scores across conditions. *Notes.* Values represent means of age- and education-corrected TMT B-A scores; error bars indicate ±1 SD. Lower corrected scores indicate a smaller B-A difference. Significance brackets and asterisks indicate Benjamini–Hochberg-adjusted Conover post-hoc comparisons (* *p* < 0.05; ** *p* < 0.01; *** p < 0.001). *Abbreviations*: TMT = Trail Making Test; B-A = TMT-B minus TMT-A; NPAB = No Physical Activity Break; OPAB = Outdoor Physical Activity Break; PABEx = Physical Activity Break with Exergame.

Condition significantly affected TMT B-A corrected scores (Friedman χ^2^(2) = 10.841, Benjamini–Hochberg-adjusted *p* = 0.005), with a small effect size (Kendall’s W = 0.129). Mean ± SD adjusted values were 96.57 ± 35.43 for NPAB, 77.71 ± 31.14 for OPAB, and 80.76 ± 30.60 for PABEx. Post-hoc Conover comparisons, interpreted after Benjamini–Hochberg correction across the full family of pairwise contrasts, indicated significantly lower TMT B-A scores for OPAB compared with NPAB (Benjamini–Hochberg-adjusted *p* = 0.009). In contrast, the differences between NPAB and PABEx (Benjamini–Hochberg-adjusted *p* = 0.110) and between OPAB and PABEx (Benjamini–Hochberg-adjusted *p* = 0.244) were not statistically significant (Figure 4).

### 3.2. Stroop Color and Word Test

**Figure 5:**
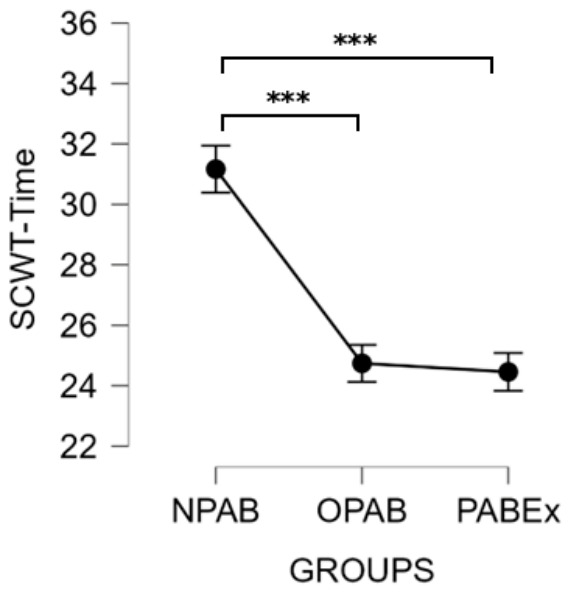
SCWT completion time across conditions. *Notes.* Values represent means of age- and education-corrected SCWT interference-time scores; error bars indicate ±1 SD. Lower corrected completion-time scores indicate better performance. Significance brackets and asterisks indicate Benjamini–Hochberg-adjusted Conover post-hoc comparisons (* *p* < 0.05; ** *p* < 0.01; *** *p* < 0.001). *Abbreviations*: SCWT = Stroop Color-Word Test; NPAB = No Physical Activity Break; OPAB = Outdoor Physical Activity Break; PABEx = Physical Activity Break with Exergame.

SCWT corrected interference-time scores differed significantly across conditions (Friedman χ^2^(2) = 44.714, Benjamini–Hochberg-adjusted *p* < 0.001), with a large condition effect (Kendall’s W = 0.532). Mean ± SD interference-corrected scores were 31.17 ± 7.35 for NPAB, 24.74 ± 5.72 for OPAB, and 24.46 ± 4.90 for PABEx. As shown in Figure 5, post-hoc Conover comparisons, interpreted after Benjamini–Hochberg correction across the full family of pairwise contrasts, showed significantly lower interference-time scores for OPAB compared with NPAB (Benjamini–Hochberg-adjusted *p* < 0.001) and for PABEx compared with NPAB (Benjamini–Hochberg-adjusted *p* < 0.001). The difference between OPAB and PABEx was not statistically significant (Benjamini–Hochberg-adjusted *p* = 0.329).

**Figure 6:**
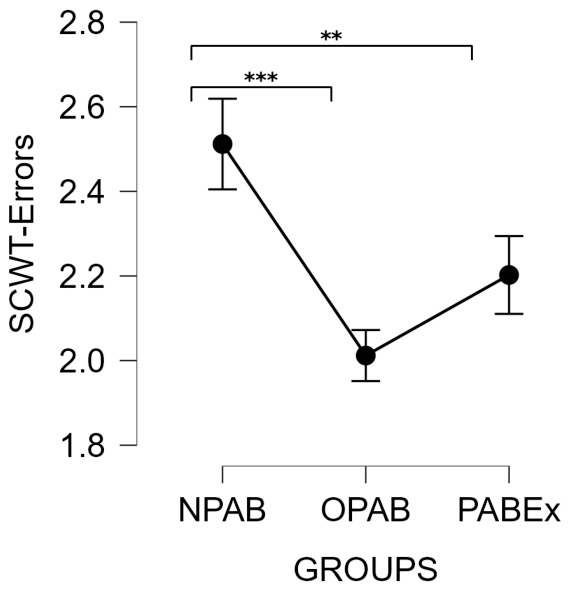
SCWT error counts across conditions. *Notes.* Values represent means of age- and education-corrected SCWT interference-error scores; error bars indicate ±1 SD. Lower corrected error scores indicate better performance. Significance brackets and asterisks indicate Benjamini–Hochberg-adjusted Conover post-hoc comparisons (* *p* < 0.05; ** *p* < 0.01; *** *p* < 0.001). *Abbreviations:* SCWT = Stroop Color-Word Test; NPAB = No Physical Activity Break; OPAB = Outdoor Physical Activity Break; PABEx = Physical Activity Break with Exergame.

SCWT corrected interference-error scores also showed a significant effect of condition (Friedman χ^2^(2) = 23.211, Benjamini–Hochberg-adjusted *p* < 0.001), with a moderate effect size (Kendall’s W = 0.276). Mean ± SD corrected error scores were 2.51 ± 0.94 for NPAB, 2.01 ± 0.49 for OPAB, and 2.20 ± 0.71 for PABEx. Post-hoc Conover comparisons, interpreted after Benjamini–Hochberg correction across the full family of pairwise contrasts, showed significantly lower corrected error scores for OPAB compared with NPAB (Benjamini–Hochberg-adjusted *p* < 0.001) and for PABEx compared with NPAB (Benjamini–Hochberg-adjusted *p* = 0.012). The difference between OPAB and PABEx was not statistically significant (Benjamini–Hochberg-adjusted *p* = 0.104) (Figure 6).

No adverse events or unintended effects were reported during any condition.

Additional analyses were conducted to examine potential period effects across the three testing sessions, irrespective of the experimental condition performed in each period. Friedman tests showed no significant differences across Period 1, Period 2, and Period 3 for TMT-A (χ^2^ = 2.096, *p* = 0.351, Kendall’s W = 0.025), TMT-B (χ^2^ = 0.299, *p* = 0.861, Kendall’s W = 0.004), TMT B-A (χ^2^ = 0.085, *p* = 0.958, Kendall’s W = 0.001), SCWT interference time (χ^2^ = 2.476, *p* = 0.290, Kendall’s W = 0.029), or SCWT interference errors (χ^2^ = 0.281, *p* = 0.869, Kendall’s W = 0.003). These findings indicate that cognitive performance did not systematically differ across the three testing periods independently of condition assignment (Table 1).

## 4. Discussion

This randomized crossover study investigated the acute effects of a 10 min physical activity break (PAB) implemented after university lectures on the cognitive function of university students, and whether these effects differed by PAB modality.

The findings generally support the hypothesis that both active-break conditions would be associated with more favorable post-condition attentional and executive performance than the passive control condition. This suggests that, after the same lecture-based academic exposure, the type of break performed was associated with measurable differences in subsequent cognitive performance. On the other hand, whereas OPAB and PABEx showed broadly comparable effects across executive outcomes, the hypothesis of a specific modality difference was not confirmed overall. TMT-A, which primarily evaluates processing speed and visuoperceptual tracking, showed the only significant between-modality difference; performance after PABEx was more favorable than performance after OPAB.

TMT-A is commonly recognized as a performance index of visual search/scanning efficiency and information processing speed, with a further visuomotor sequencing component [39]. In the current study, both PAB conditions were associated with faster TMT-A performance when compared to NPAB. This suggests that a short period of movement immediately after the full lecture period, which is accompanied by sustained cognitive engagement and continuous allocation of attentional resources, may be associated with more efficient acute speeded visual-attentional processes.

Information processing speed is among the cognitive domains most sensitive to acute bouts of physical activity [40]. Brief periods of outdoor physical activity, such as moderate walking, have been shown to facilitate processing speed and visual scanning efficiency [41], likely through transient increases in physiological arousal, cerebral blood flow, and catecholaminergic activity, which collectively enhance neural efficiency in attentional networks [42,43,44]. Outdoor activity may provide further acute cognitive support by decreasing cognitive fatigue accrued from prolonged sedentary activities, allowing for faster perceptual encoding and reaction execution [45,46].

Studies conducted in an educational context suggest that interrupting prolonged sitting with short bouts of outdoor movement is associated with more favorable acute speeded attentional performance and reaction time, particularly when tasks emphasize rapid visuoperceptual processing rather than higher-order executive coordination [47,48,49].

Beyond the effects attributable to physical activation alone, exergame-based activity may involve task-related features that are relevant to processing speed, including bodily movement, visuospatial engagement, and rapid stimulus-response mapping [50,51,52]. Exergames require sustained visual monitoring, rapid target discrimination, and immediate motor responses, thereby partly overlapping with the cognitive operations underlying speeded attentional tasks (e.g., visual scanning, perceptual tracking, and response execution) [53]. Studies have shown that cognitively engaging physical activity can enhance processing speed more effectively than movements with minimal cognitive demands, particularly in young adults and adolescents [50,54]. Similarly, a meta-analysis of active video games suggests that exergaming can produce acute improvements in processing speed and attentional efficiency, likely due to the concurrent activation of sensorimotor and attentional systems [55]. Exergaming may amplify the effects of brief physical activity on processing speed by simultaneously stimulating arousal-related mechanisms and task-relevant attentional processes [56,57]. In line with this evidence, the task characteristics of PABEx may have contributed to the more favorable TMT-A performance observed relative to OPAB, although this modality-specific pattern should be considered within the broader result that both active-break conditions were associated with more favorable cognitive performance than the passive control condition.

The results of the TMT-B and the Stroop test are generally regarded as more significant indicators of executive function than the TMT-A, as both require continuous coordination of control processes rather than predominantly rapid visual scanning [58,59]. Specifically, TMT-B requires substantial demands on alternating attention, task switching, and cognitive flexibility, as performance depends on maintaining two mental sets and efficiently shifting between them [36]; Stroop interference performance reflects interference control and inhibitory control, requiring suppression of a prepotent response in favor of a task-relevant one [60]. In this study, both OPAB and PABEx were associated with more favorable post-condition TMT-B and Stroop interference indices relative to NPAB, which is consistent with meta-analytic evidence indicating that a single brief bout of physical activity can be associated with acute facilitation of executive-task performance, with particularly reliable effects when assessment occurs shortly after the activity and when tasks index inhibition or related control operations [61,62].

Acute activity is associated with transient changes in arousal and neurophysiological state that can facilitate prefrontal control processes, which are central to task switching, alternating attention, and interference control [63]. Research grounded in Attention Restoration Theory suggests that exposure to natural environments can replenish directed attention resources after cognitive effort, with experimental evidence showing that interacting with nature (including walking in natural settings) can improve executive attention and related control functions [64]. Although the strength and consistency of “nature effects” can vary across paradigms and samples, meta-analytic research has addressed this heterogeneity and found that environment may contribute meaningfully to cognitive restoration, particularly for attention-related components related to executive control efficiency [65,66]. Related evidence further suggests that outdoor and technology-mediated exercise modalities may elicit different psychophysiological and experiential responses, including heart rate, enjoyment, mindfulness, motivation, and intention to engage in green exercise [67,68]. OPAB may be especially suited to supporting acute executive-task performance after a lengthy lecture since it combines moderate movement with an environmental context that may reduce cognitive fatigue and support post-activity control resource availability, thereby facilitating the flexible coordination demands that characterize tasks such as TMT-B and Stroop interference performance [46].

Since the game requires regular movements of the whole body or upper limbs to interact with a constantly shifting and cognitively challenging virtual environment, exergaming offers a condition in which physical activation is coupled with continuous perceptual, attentional, and motor demands [50]. Active video games’ physical demands may induce typical neuronal pathways associated with physical activity, such as increased production and release of neurotrophic and growth factors, which in turn are thought to support neurobiological conditions relevant to cognitive functioning [69]. In the context of acute exergame-based activity, these processes may contribute to a broader state of cognitive readiness, particularly when combined with the attentional and visuomotor demands of the task.

The cognitively enriched nature of exergames may directly engage executive control operations in addition to the physiological activation contribution. Participants must continuously regulate action initiation and inhibition, sustain and shift attention, integrate sensory signals, plan motor sequences, make immediate choices, and respond accurately under time pressure [31]. These demands are consistent with evidence suggesting that cognitively enriched physical activity may be particularly relevant for inhibitory control and other executive processes [70], and with findings linking cognitively engaging video game play to neural systems involved in visuospatial processing, visuomotor integration, and motor planning/execution [71].

Furthermore, as active video games combine cognitive engagement with exercise-related neurobiological processes and cognitively demanding task features relevant to executive control, these overlapping lines of evidence provide a plausible framework for synergistic or additive effects of exergaming on executive task performance [72]. At the same time, for higher-order control tasks, the absence of consistent differences between OPAB and PABEx may indicate that the two active conditions shared sufficient activation-related features to support executive-task performance, despite differing in modality-specific cognitive demands [50,73]. Meta-analytic and individual participant data meta-analytic research support the view that acute exercise may improve executive performance by inducing transient changes in arousal and neuromodulatory activity, and that heterogeneity in findings is often due to moderators such as baseline performance and task demands rather than exercise “type” itself [61,74].

Once a short bout of activity is sufficient to shift participants from a post-lecture state characterized by high attentional expenditure and incipient cognitive fatigue toward a more optimal activation state, additional modality-specific features (e.g., the richer visuomotor demands of exergaming) may offer limited incremental value on outcomes where set maintenance, switching efficiency, and conflict resolution dominate performance constraints [61,62]. A second, complementary interpretation is that PABEx, while cognitively engaging, may impose its own concurrent control demands during the break (rapid selection and response programming), reducing the likelihood that it will outperform a rhythmically, comparatively automated OPAB on executive indices reflecting control resource availability and coordination immediately after the break [40,75].

Crucially, however, when executive demands were examined through the TMT B-A difference score, OPAB differed from NPAB, whereas PABEx did not. The interpretive value of B-A stems from the rationale that subtracting A from B is intended to partially reduce baseline processing speed and shared visuomotor demands, thereby providing a derived index of the additional demands involved in Part B [59]. However, because TMT B-A remains a difference score, it should not be considered a process-pure measure of cognitive flexibility, as residual variance related to processing speed, visual scanning, and task-execution demands may still contribute to performance. Outdoor walking may provide physical activation with relatively limited concurrent task demands, thereby supporting subsequent performance on tasks requiring executive coordination [76]. In contrast, the exergame requires continuous selection, rapid visuomotor adjustments, and sustained engagement, potentially engaging cognitive-control processes during the break itself [72]. Such engagement may be advantageous for speeded attentional tasks, as observed in TMT-A, but may not necessarily translate into additional benefit on a derived and composite index such as TMT B-A. Meta-analytic research suggests that acute exercise effects are moderated by task type and timing; facilitation tends to be more consistent for speeded tasks and may be less robust, or more sensitive to parameters, for higher-order coordination metrics [74]. This executive component may be less malleable in the very short term or may require a more precise matching of intensity and cognitive engagement than was achievable with the present modalities. Overall, the TMT B-A findings should therefore be interpreted as a small modality-specific difference in a composite measure of speeded executive performance, rather than as definitive evidence of a selective improvement in cognitive flexibility.

In terms of the duration and intensity of acute active-break interventions, research from secondary-school samples indicates that favorable acute cognitive responses are most consistently reported when breaks last approximately 4–10 min and are provided at moderate-to-high intensity [77,78]. In university settings, the most implemented durations range from 5 to 15 min [79,80], and moderate-to-vigorous intensities have generally been associated with more favorable acute cognitive performance.

Despite the novelty of this study in terms of duration, comparative design, and university setting, several limitations should be acknowledged that could be addressed in future research.

Because the study focused exclusively on acute effects, the findings cannot be generalized to long-term or cumulative cognitive benefits of repeated PAB exposure.

Although the crossover design mitigates between-subject variability, the sample size, while adequately powered for within-subject analyses, may have limited the ability to detect small between-modality differences, particularly on executive indices where effects are typically modest in high-functioning young adults. A further limitation is that exercise intensity was not monitored through heart rate, RPE, or other physiological markers. Therefore, although OPAB and PABEx were procedurally standardized, their physiological dose may have differed. This limits the interpretation of modality-specific effects, particularly the PABEx advantage on TMT-A, which may reflect differences in arousal or cardiovascular activation in addition to the cognitive-motor demands of the exergame. Future studies should quantify and, where possible, match exercise intensity across active-break modalities. Although participants received standardized pre-session instructions regarding sleep routine, physical activity, caffeine, stimulant intake, and breakfast habits, these factors were not objectively monitored or formally recorded. Therefore, residual variability in sleep duration, time since breakfast, nicotine or stimulant use, and individual familiarity with Fruit Ninja or exergaming may have influenced acute cognitive performance or engagement during the PABEx condition.

Although additional analyses showed no evidence of significant period effects across the three testing sessions, formal sequence-effect analyses were not conducted. Therefore, residual order-related influences cannot be entirely excluded, despite the randomized and counterbalanced crossover design and the 7-day washout interval between sessions.

Moreover, the study did not include subjective measures of enjoyment, perceived fatigue, mood, or affective state, which would have helped clarify whether the different experiential profiles of OPAB and PABEx contributed to the observed cognitive responses. In addition, previous familiarity with Fruit Ninja Kinect or exergaming was not assessed, which may have contributed to individual variability in movement execution, task engagement, and cognitive load during the PABEx condition. Another limitation concerns the lack of baseline assessment of habitual physical activity levels and sedentary behavior. Although all participants completed a standardized lecture-based sedentary period before each experimental condition, the study did not include self-report or device-based measures to characterize participants as habitually sedentary, moderately active, or highly active. This limits the ecological characterization of the sample and prevents examination of whether habitual activity or sedentary profiles moderated the acute cognitive response to the PAB conditions. Although efforts were made to standardize testing conditions, residual influences related to motivation, prior fatigue, or individual differences in familiarity with exergaming cannot be fully excluded. Finally, the study was retrospectively registered at ClinicalTrials.gov, which should be considered when evaluating protocol transparency and the exploratory nature of the trial.

## 5. Conclusions

This randomized crossover study showed that, after a standardized lecture-based academic activity, post-condition attentional and executive performance differed according to break modality. Both active modalities, OPAB and PABEx, were associated with more favorable cognitive outcomes than passive seated rest, indicating that brief active breaks may represent a useful strategy to support cognitive efficiency in university students after prolonged lecture-based sedentary activity.

These findings highlight the potential usefulness of incorporating brief physical activity breaks into university timetables as an approach for improving cognitive efficiency during academically intensive periods. Given their brevity and versatility, outdoor and exergame-based PABs may represent useful, low-cost strategies to support students’ attentional efficiency and executive control without interfering with their learning time. Given that early adulthood is a key period for enhancing cognitive development, future research should investigate long-term implementation, dose-response relationships, and individual or environmental characteristics that may improve the cognitive impact of different PAB modalities in higher education settings. In this direction, future studies should also include objective and subjective indicators of exercise intensity in order to better characterize the physiological dose of outdoor and exergame-based breaks and refine the interpretation of modality-specific effects.

## Figures and Tables

**Table 1:** Period-effect analysis across the three testing sessions.

Cognitive Outcome	χ^2^	*p*-Value	Kendall’s W
TMT-A	2.096	0.351	0.025
TMT-B	0.299	0.861	0.004
TMT B-A	0.085	0.958	0.001
SCWT interference time	2.476	0.290	0.029
SCWT interference errors	0.281	0.869	0.003

*Notes.* Period effects were examined using Friedman tests comparing cognitive outcomes across Period 1, Period 2, and Period 3, irrespective of the experimental condition performed in each period. Kendall’s W is reported as the effect-size estimate. *Abbreviations:* TMT-A = Trail Making Test-Part A; TMT-B = Trail Making Test-Part B; TMT B-A = TMT-B minus TMT-A; SCWT = Stroop Color-Word Test; χ^2^ = chi-square statistic; Kendall’s W = Kendall’s coefficient of concordance.

## Data Availability

The data presented in this study are available upon request from the first author. The data are not publicly available due to privacy concerns.

## Supplementary Materials

The following supporting information can be downloaded at: https://www.mdpi.com/article/10.3390/healthcare14132010/s1, Table S1: Completed CONSORT checklist for randomized crossover trials.
