# Supplementary material for: Acute Cognitive Effects of Brief Physical Activity Breaks After Lecture-Based Academic Activity in Undergraduate University Students: A Randomized Crossover Study
Source: Healthcare (Basel). 2026 Jul 6;14(13):2010. doi: 10.3390/healthcare14132010 (PMC13361387; doi:10.3390/healthcare14132010)
Supplement: Supplementary file 1 [file healthcare-14-02010-s001.zip › healthcare-4372089-supplementary.pdf]

CONSORT Checklist for Randomised Crossover Trials

Adapted for: Pepe et al. - Acute Cognitive Effects of Brief Physical Activity Breaks After Lecture-Based Academic Activity in Undergraduate University Students: A Randomized Crossover Study

Source guideline: Dwan K, Li T, Altman DG, Elbourne D. CONSORT 2010 statement: extension to randomised crossover trials. BMJ. 2019;366:l4378. doi:10.1136/bmj.l4378.

Note: The CONSORT crossover extension modifies the standard CONSORT checklist for crossover designs. Page numbers/locations should be checked against the final submitted manuscript after formatting.

Section/topic	Item No.	CONSORT crossover item	Reported on page/section
Title	1a	Identification as a randomised crossover trial in the title.	Title
Abstract	1b	Specify a crossover design and report all information outlined for the abstract.	Abstract
Introduction - Background	2a	Scientific background and explanation of rationale.	Introduction
Introduction - Objectives	2b	Specific objectives or hypotheses.	End of Introduction
Methods - Trial design	3a	Rationale for a crossover design. Description of the design features, including allocation ratio, especially the number and duration of periods, duration of washout period, and consideration of carry-over effect.	Methods: Participants and study design; Setting and Procedure
Methods - Changes from protocol	3b	Important changes to methods after trial commencement, such as eligibility criteria, with reasons.	No changes after trial commencement.
Methods - Participants	4a	Eligibility criteria for participants.	Methods: Participants and study design
Methods - Settings and location	4b	Settings and locations where the data were collected.	Methods: Participants and study design; Setting and Procedure
Methods - Interventions	5	The interventions with sufficient details to allow replication, including how and when they were actually administered.	Methods: Physical Activity Break Interventions
Methods - Outcomes	6a	Completely defined prespecified primary and secondary outcome measures, including how and when they were assessed.	Methods: Cognitive Assessment; Statistical Analysis
Methods - Changes to outcomes	6b	Any changes to trial outcomes after the trial commenced, with reasons.	No changes after trial commencement.
Methods - Sample size	7a	How sample size was determined, accounting for within-participant variability.	Methods: Participants and study design
Methods - Interim analyses and stopping guidelines	7b	When applicable, explanation of any interim analyses and stopping guidelines.	Not applicable
Randomisation - Sequence generation	8a	Method used to generate the random allocation sequence.	Methods: Setting and Procedure
Randomisation - Type	8b	Type of randomisation; details of any restriction such as blocking and block size.	Methods: Setting and Procedure
Randomisation - Allocation concealment	9	Mechanism used to implement the random allocation sequence, describing any steps taken to conceal the sequence until interventions were assigned.	Methods: Setting and Procedure
Randomisation - Implementation	10	Who generated the random allocation sequence, who enrolled participants, and who assigned participants to the sequence of interventions.	Methods: Setting and Procedure
Blinding	11a	If done, who was blinded after assignment to interventions, for example participants, care providers, those assessing outcomes, and how.	Methods: Setting and Procedure
Similarity of interventions	11b	If relevant, description of the similarity of interventions.	Not applicable / partly Methods: Interventions
Methods - Statistical methods	12a	Statistical methods used to compare groups for primary and secondary outcomes that are appropriate for crossover design, that is, based on within-participant comparison.	Methods: Statistical Analysis
Methods - Additional analyses	12b	Methods for additional analyses, such as subgroup analyses and adjusted analyses.	Methods: Statistical Analysis

Section/topic	Item No.	CONSORT crossover item	Reported on page/section
Results - Participant flow	13a	The numbers of participants who were randomly assigned, received intended treatment, and were analysed for the primary outcome, separately for each sequence and period. A diagram is strongly recommended.	CONSORT crossover flow diagram; Results/Methods
Results - Losses and exclusions	13b	Number of participants excluded at each stage, with reasons, separately for each sequence and period.	CONSORT crossover flow diagram
Results - Recruitment	14a	Dates defining the periods of recruitment and follow-up.	Methods: Participants and study design
Results - Trial end	14b	Why the trial ended or was stopped.	Not applicable; The trial ended as planned after completion of all three crossover periods.
Results - Baseline data	15	A table showing baseline demographic and clinical characteristics by sequence and period.	Overall sample characteristics are reported in Methods; no separate baseline table by sequence was included.
Results - Numbers analysed	16	Number of participants (denominator) included in each analysis and whether the analysis was by original assigned groups.	Results; Statistical Analysis
Results - Outcomes and estimation	17a	For each primary and secondary outcome, results including estimated effect size and precision should be based on within-participant comparisons. In addition, results for each intervention in each period are recommended.	Results; Figures
Results - Binary outcomes	17b	For binary outcomes, presentation of both absolute and relative effect sizes is recommended.	Not applicable
Results - Ancillary analyses	18	Results of any other analyses performed, including subgroup analyses and adjusted analyses, distinguishing prespecified from exploratory.	Not applicable
Results - Harms	19	Describe all important harms or unintended effects in a way that accounts for the crossover design.	Results: end of Section 3.2
Discussion - Limitations	20	Trial limitations, addressing sources of potential bias, imprecision, and, if relevant, multiplicity of analyses. Consider potential carry-over effects.	Discussion: Limitations
Discussion - Generalisability	21	Generalisability, external validity, applicability of the trial findings.	Discussion
Discussion - Interpretation	22	Interpretation consistent with results, balancing benefits and harms, and considering other relevant evidence.	Discussion; Conclusions
Other information - Registration	23	Registration number and name of trial registry.	ClinicalTrials.gov, NCT07624084; retrospectively registered on 28 May 2026. Reported in Abstract and Methods.
Other information - Protocol	24	Where the full trial protocol can be accessed, if available.	Not available
Other information - Funding	25	Sources of funding and other support, such as supply of drugs, and role of funders.	Funding statement

Legend: Modified items in the crossover extension particularly affect design rationale, sample size, statistical methods, participant flow, baseline data, outcomes/estimation, harms, and limitations. For this three-condition, three-period trial, the flow diagram should be adapted to NPAB, OPAB and PABEx across Periods 1-3.
